# Supplementary material for: Seminoma and Embryonal Carcinoma Footprints Identified by Analysis of Integrated Genome-Wide Epigenetic and Expression Profiles of Germ Cell Cancer Cell Lines
Source: PLoS One. 2014 Jun 2;9(6):e98330. doi: 10.1371/journal.pone.0098330 (PMC4041891; doi:10.1371/journal.pone.0098330)
Supplement: File S1 — ZIP file containing DMRforPairs output for significant regions. Please start from the html files. (ZIP) [file pone.0098330.s008.zip › figures/3884.pdf]

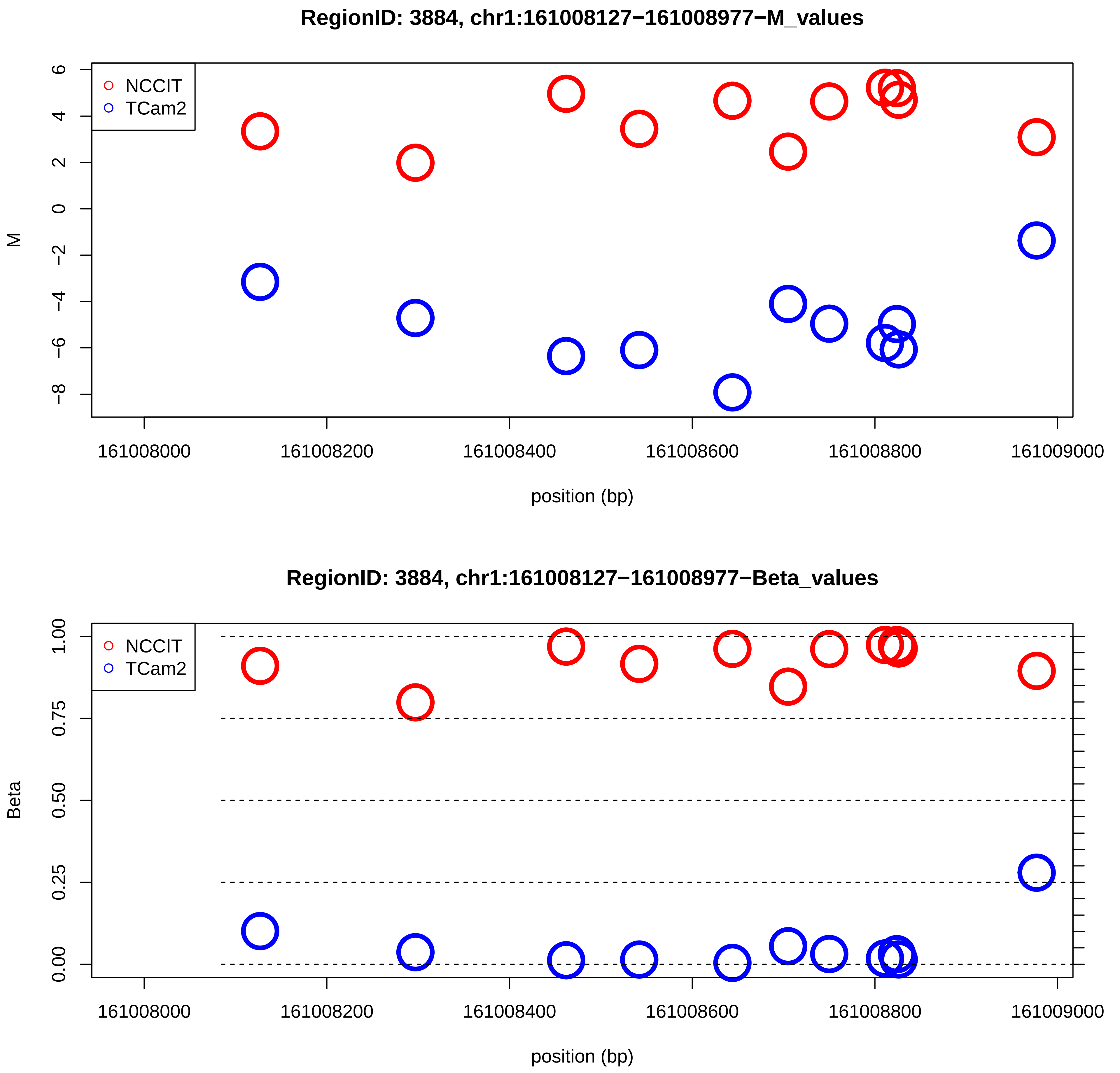
RegionID: 3884, chr1:161008127−161008977−M_values
NCCIT
TCam2
M
position (bp)
161008000
161008200
161008400
161008600
161008800
161009000
RegionID: 3884, chr1:161008127−161008977−Beta_values
Beta
0.00
0.25
0.50
0.75
1.00

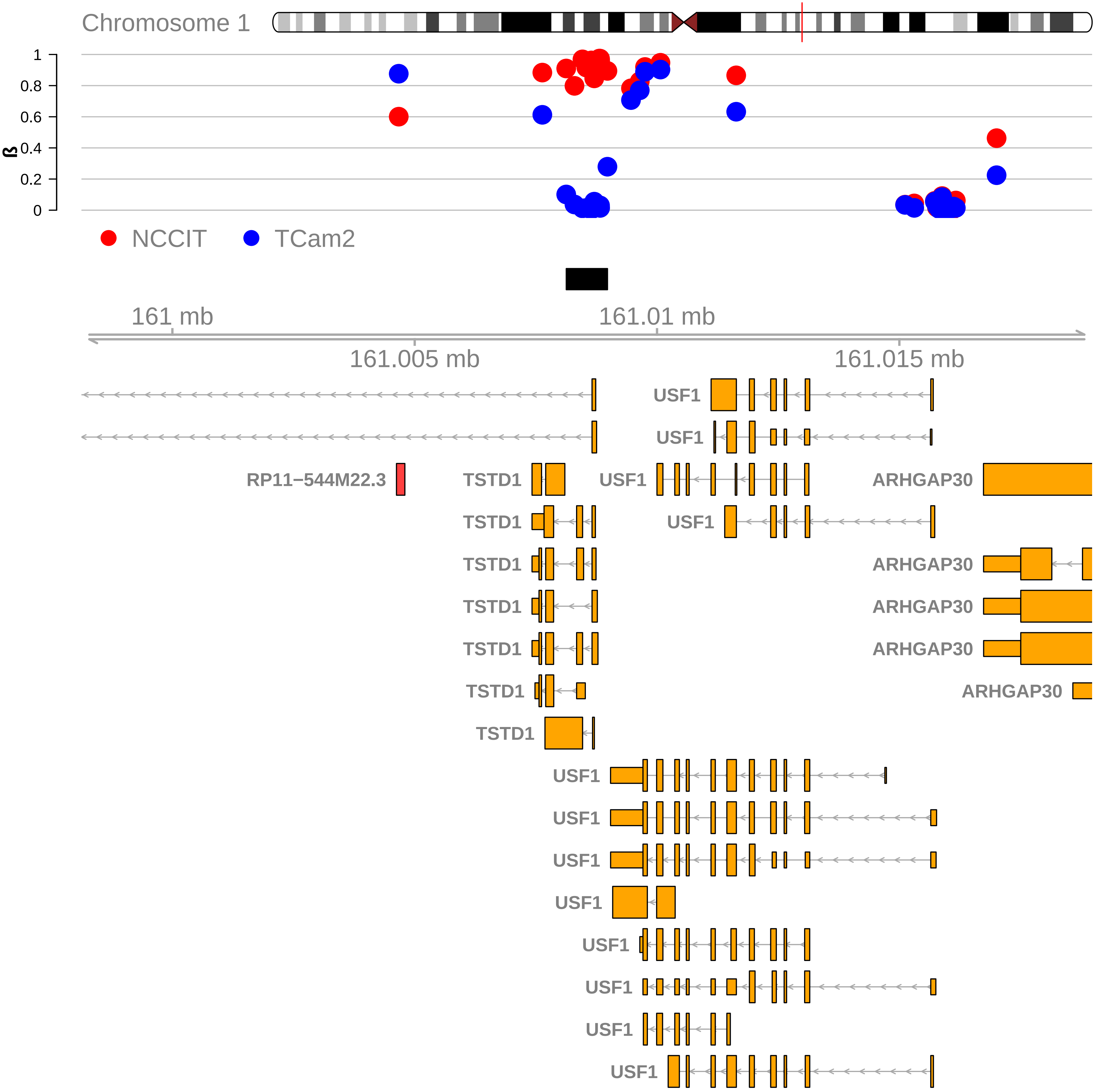

Chromosome 1
β
1
0.8
0.6
0.4
0.2
0
NCCIT
TCam2
161 mb
161.005 mb
161.01 mb
161.015 mb
USF1
RP11−544M22.3
TSTD1
ARHGAP30
